# *N*-Benzoyl-*N*′-(2-chloro-3-pyrid­yl)thio­urea

**DOI:** 10.1107/S1600536809027081

**Published:** 2009-07-18

**Authors:** Yu-Jie Ding, Jian Yao, Jian-Chao Wu, Wen-Kui Dong, Jun-Feng Tong

**Affiliations:** a Department of Biochemical Engineering, Anhui University of Technology and Science, Wuhu 241000, People’s Republic of China; b School of Chemical and Biological Engineering, Lanzhou Jiaotong University, Lanzhou 730070, People’s Republic of China

## Abstract

The title compound, C_13_H_10_ClN_3_OS, was prepared by the reaction of 3-amino-2-chloropyridine with benzoyl isothio­cyanate at room temperature. The thio­urea group makes dihedral angles of 47.17 (5) and 51.88 (4)°, respectively, with the benzene and pyridyl rings, while the angle between the benzene and pyridine rings is 8.91 (3)°. Inter­molecular hydrogen-bond inter­actions link neighbouring mol­ecules into an infinite supra­molecular structure.

## Related literature

For the biological activities of benzanilide and its N-substituted derivatives, see: Teoh *et al.* (1999 ▶); Campo *et al.* (2002 ▶). For the functions of related chloro­phenyl compounds, see: Saeed *et al.* (2008 ▶); Gowda *et al.* (2008*a* ▶,*b* ▶,*c* ▶). For an isomeric compound, see: Chai *et al.* (2008 ▶). For our previous work on thio­urea and its derivatives, see: Dong *et al.* (2006 ▶, 2007 ▶, 2008*a* ▶,*b* ▶). For the synthetic procedure, see: Ding *et al.* (2008 ▶).
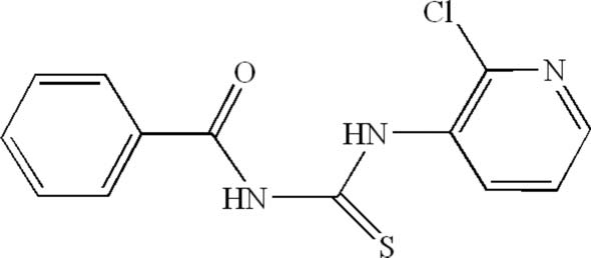


## Experimental

### 

#### Crystal data

C_13_H_10_ClN_3_OS
*M* *_r_* = 291.73
Monoclinic, 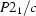

*a* = 3.9443 (4) Å
*b* = 14.9250 (15) Å
*c* = 22.268 (2) Å
β = 93.889 (1)°
*V* = 1307.9 (2) Å^3^
*Z* = 4
Mo *K*α radiation
μ = 0.45 mm^−1^
*T* = 298 K
0.41 × 0.20 × 0.18 mm

#### Data collection

Bruker SMART 1000 CCD area-detector diffractometer
Absorption correction: multi-scan (*SADABS*; Sheldrick, 1996 ▶) *T*_min_ = 0.839, *T*_max_ = 0.924
6459 measured reflections
2315 independent reflections
1661 reflections with *I* > 2σ(*I*)
*R*_int_ = 0.040

#### Refinement

*R*[*F* ^2^ > 2σ(*F* ^2^)] = 0.038
*wR*(*F* ^2^) = 0.088
*S* = 1.03
2315 reflections
172 parameters
H-atom parameters constrained
Δρ_max_ = 0.26 e Å^−3^
Δρ_min_ = −0.21 e Å^−3^

### 

Data collection: *SMART* (Siemens, 1996 ▶); cell refinement: *SAINT* (Siemens, 1996 ▶); data reduction: *SAINT*; program(s) used to solve structure: *SHELXS97* (Sheldrick, 2008 ▶); program(s) used to refine structure: *SHELXL97* (Sheldrick, 2008 ▶); molecular graphics: *SHELXTL* (Sheldrick, 2008 ▶); software used to prepare material for publication: *SHELXTL*.

## Supplementary Material

Crystal structure: contains datablocks global, I. DOI: 10.1107/S1600536809027081/at2841sup1.cif

Structure factors: contains datablocks I. DOI: 10.1107/S1600536809027081/at2841Isup2.hkl

Additional supplementary materials:  crystallographic information; 3D view; checkCIF report

## Figures and Tables

**Table 1:** Hydrogen-bond geometry (Å, °)

*D*—H⋯*A*	*D*—H	H⋯*A*	*D*⋯*A*	*D*—H⋯*A*
N2—H2⋯O1	0.86	1.94	2.633 (2)	137
N1—H1⋯S1^i^	0.86	2.74	3.5982 (18)	178
C12—H12⋯O1^ii^	0.93	2.70	3.324 (3)	125

Symmetry codes: (i) 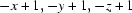; (ii) 
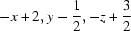
.

**Fig. 1.**
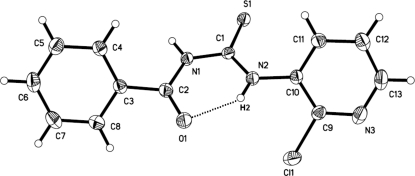
The molecular structure of the title compound with atom numbering scheme. Displacement ellipsoids for non-hydrogen atoms are drawn at the 30% probability level.

**Fig. 2.**
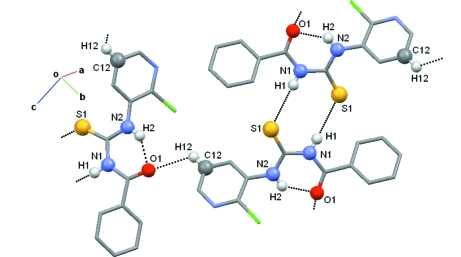
Part of the supramolecular structure of the title compound. Intramolecular and intermolecular hydrogen bonds of the title compound are shown as dashed lines.

## supplementary crystallographic information

### Comment

Benzanilide and its N-substituted derivatives have been considered to be a class of privileged structural compounds, which usually have excellent biological activities (Teoh *et al.*, 1999; Campo *et al.*, 2002). However, the literatures are full of the function of the 2-chloro-4-nitrophenyl (Saeed *et al.*, 2008), 3,5-dichlorophenyl (Gowda *et al.*, 2008*a*) and 3-chlorophenyl (Gowda *et al.*, 2008*b*; Gowda *et al.*, 2008*c*) and also structures of benzamide and related compounds. As an extension of our work (Dong *et al.*, 2006; Dong *et al.*, 2007; Dong *et al.*, 2008*a*; Dong *et al.*, 2008*b*) on synthesis and structural characterization of thiourea and its derivatives, here report the synthesis and structure of the title compound.

In the molecule of the title compound, *N*-benzoyl-*N*'-(2-chloro-3-pyridyl)thiourea (Fig. 1), which is isomeric compound to its observed in the structures of *N*-(2-chlorobenzoyl)-*N*'-(3-pyridyl)thiourea (Chai *et al.*, 2008). The thiourea group makes dihedral angles of 47.17 (5)° and 51.88 (4)° with the benzene and pyridyl rings respectively, while the angle between the benzene and pyridine rings is 8.91 (3)°. The carbonyl group forms an intramolecular hydrogen bond with the N2—H2 group, which forms a six-membered ring (C2/N1/C1/N2/H2/O1) structure, the H2···O1 bond length is 1.94 Å. The C═O bond length with 1.218 (3) Å is longer than the average C═O bond length [1.200 Å], which is due to intramolecular hydrogen bonding. This is similar to the situation found in the structure of *N*-benzoyl-*N*'-(3-pyridyl)thiourea (Dong *et al.*, 2006). The crystal structure is further stabilized by intermolecular N1—H1···S1 and C12—H12···O1 hydrogen bonds interactions (Table 1, Fig. 2), which link neighbouring molecules into an infinite supramolecular structure.

### Experimental

*N*-Benzoyl-*N*'-(2-chloro-3-pyridyl)thiourea was synthesized according to an analogous method reported earlier (Ding *et al.*, 2008). Benzoyl chloride (702.8 mg, 5.00 mmol) was reacted with ammonium thiocyanate (380.6 mg, 5.00 mmol) in acetonitrile solution (25 ml) continuring stirring for 3 h at room temperature, to give the corresponding benzoyl isothiocyanate, which was added 3-amino-2-chloropyrldine (642.8 mg, 5.00 mmol). After stirring for 20 h at room temperature, the precipitate was reduced pressure filtered, washed successively with acetonitrile and diethyl ether. The product was dried *in vacuo*, and obtained 599.2 mg of needle-like crystalline solid. Yield, 41.07%. m.p. 424–426 K. Colorless single crystals suitable for X-ray diffraction studies were obtained after two weeks by slow evaporation from a mixture of ethyl acetate/acetone (1:1) of *N*-benzoyl-*N*'-(2-chloro-3-pyridyl)thiourea at room temperture. Analysis calculated for C_13_H_10_ClN_3_OS (%): C 53.52, H 3.45, N 14.40. Found: C 53.61, H 3.51, N 14.3.

### Refinement

H atoms were treated as riding atoms with distances C—H = 0.93 Å (CH), N—H = 0.86 Å, and *U*_iso_(H) = 1.2*U*_eq_(C,N).

### Crystal data

**Table:**

C_13_H_10_ClN_3_OS	*F*(000) = 600
*M**_r_* = 291.73	*D*_x_ = 1.482 Mg m^−^^3^
Monoclinic, *P*2_1_/*c*	Mo *K*α radiation, λ = 0.71073 Å
Hall symbol: -P 2ybc	Cell parameters from 2087 reflections
*a* = 3.9443 (4) Å	θ = 2.3–25.0°
*b* = 14.9250 (15) Å	µ = 0.45 mm^−^^1^
*c* = 22.268 (2) Å	*T* = 298 K
β = 93.889 (1)°	Needle-like, colourless
*V* = 1307.9 (2) Å^3^	0.41 × 0.20 × 0.18 mm
*Z* = 4	

### Data collection

**Table:**

Bruker SMART 1000 CCD area-detector diffractometer	2315 independent reflections
Radiation source: fine-focus sealed tube	1661 reflections with *I* > 2σ(*I*)
graphite	*R*_int_ = 0.040
φ and ω scans	θ_max_ = 25.0°, θ_min_ = 1.6°
Absorption correction: multi-scan (*SADABS*; Sheldrick, 1996)	*h* = −4→4
*T*_min_ = 0.839, *T*_max_ = 0.924	*k* = −17→14
6459 measured reflections	*l* = −26→24

### Refinement

**Table:**

Refinement on *F*^2^	Primary atom site location: structure-invariant direct methods
Least-squares matrix: full	Secondary atom site location: difference Fourier map
*R*[*F*^2^ > 2σ(*F*^2^)] = 0.038	Hydrogen site location: inferred from neighbouring sites
*wR*(*F*^2^) = 0.088	H-atom parameters constrained
*S* = 1.03	*w* = 1/[σ^2^(*F*_o_^2^) + (0.0355*P*)^2^ + 0.2536*P*] where *P* = (*F*_o_^2^ + 2*F*_c_^2^)/3
2315 reflections	(Δ/σ)_max_ < 0.001
172 parameters	Δρ_max_ = 0.26 e Å^−^^3^
0 restraints	Δρ_min_ = −0.21 e Å^−^^3^

### Special details

**Table:**

Geometry. All e.s.d.'s (except the e.s.d. in the dihedral angle between two l.s. planes) are estimated using the full covariance matrix. The cell e.s.d.'s are taken into account individually in the estimation of e.s.d.'s in distances, angles and torsion angles; correlations between e.s.d.'s in cell parameters are only used when they are defined by crystal symmetry. An approximate (isotropic) treatment of cell e.s.d.'s is used for estimating e.s.d.'s involving l.s. planes.
Refinement. Refinement of *F*^2^ against ALL reflections. The weighted *R*-factor *wR* and goodness of fit *S* are based on *F*^2^, conventional *R*-factors *R* are based on *F*, with *F* set to zero for negative *F*^2^. The threshold expression of *F*^2^ > σ(*F*^2^) is used only for calculating *R*-factors(gt) *etc*. and is not relevant to the choice of reflections for refinement. *R*-factors based on *F*^2^ are statistically about twice as large as those based on *F*, and *R*- factors based on ALL data will be even larger.

### Fractional atomic coordinates and isotropic or equivalent isotropic displacement parameters (Å^2^)

**Table:**

	*x*	*y*	*z*	*U*_iso_*/*U*_eq_	
S1	0.45798 (17)	0.45341 (4)	0.58736 (2)	0.0389 (2)	
Cl1	0.4376 (2)	0.65386 (5)	0.77102 (3)	0.0581 (2)	
N1	0.6989 (5)	0.61464 (12)	0.56704 (8)	0.0351 (5)	
H1	0.6662	0.5986	0.5300	0.042*	
N2	0.7371 (5)	0.57099 (12)	0.66633 (7)	0.0380 (5)	
H2	0.8131	0.6244	0.6727	0.046*	
N3	0.5774 (6)	0.49783 (16)	0.81887 (9)	0.0523 (6)	
O1	0.8401 (6)	0.73435 (11)	0.62683 (7)	0.0629 (6)	
C1	0.6406 (6)	0.54897 (14)	0.60938 (9)	0.0313 (5)	
C2	0.8025 (7)	0.70230 (15)	0.57650 (10)	0.0387 (6)	
C3	0.8633 (6)	0.75438 (14)	0.52169 (10)	0.0337 (6)	
C4	1.0013 (6)	0.71616 (16)	0.47233 (10)	0.0374 (6)	
H4	1.0520	0.6553	0.4724	0.045*	
C5	1.0642 (7)	0.76769 (17)	0.42297 (11)	0.0475 (7)	
H5	1.1643	0.7420	0.3905	0.057*	
C6	0.9790 (7)	0.85723 (18)	0.42174 (12)	0.0541 (8)	
H6	1.0176	0.8917	0.3881	0.065*	
C7	0.8371 (7)	0.89566 (17)	0.47016 (12)	0.0534 (8)	
H7	0.7760	0.9558	0.4688	0.064*	
C8	0.7846 (7)	0.84549 (16)	0.52092 (11)	0.0459 (7)	
H8	0.6974	0.8723	0.5543	0.055*	
C9	0.5921 (6)	0.54468 (16)	0.76902 (10)	0.0386 (6)	
C10	0.7247 (6)	0.51388 (15)	0.71689 (9)	0.0331 (6)	
C11	0.8605 (7)	0.42887 (16)	0.71795 (11)	0.0415 (6)	
H11	0.9587	0.4060	0.6844	0.050*	
C12	0.8487 (7)	0.37809 (17)	0.76953 (11)	0.0488 (7)	
H12	0.9360	0.3202	0.7713	0.059*	
C13	0.7047 (8)	0.41517 (19)	0.81819 (12)	0.0547 (8)	
H13	0.6955	0.3805	0.8527	0.066*	

### Atomic displacement parameters (Å^2^)

**Table:**

	*U*^11^	*U*^22^	*U*^33^	*U*^12^	*U*^13^	*U*^23^
S1	0.0473 (4)	0.0371 (4)	0.0322 (3)	−0.0108 (3)	0.0018 (3)	−0.0030 (3)
Cl1	0.0701 (5)	0.0542 (4)	0.0505 (4)	0.0118 (4)	0.0069 (4)	−0.0115 (3)
N1	0.0486 (13)	0.0312 (11)	0.0250 (10)	−0.0073 (10)	−0.0004 (9)	−0.0021 (8)
N2	0.0557 (14)	0.0300 (11)	0.0279 (10)	−0.0078 (10)	−0.0009 (9)	−0.0024 (8)
N3	0.0615 (17)	0.0609 (16)	0.0348 (12)	−0.0110 (13)	0.0049 (11)	0.0027 (11)
O1	0.1150 (19)	0.0399 (10)	0.0337 (10)	−0.0175 (11)	0.0031 (10)	−0.0068 (8)
C1	0.0322 (14)	0.0317 (12)	0.0301 (12)	0.0023 (11)	0.0040 (10)	−0.0022 (10)
C2	0.0478 (17)	0.0328 (14)	0.0352 (14)	−0.0048 (12)	0.0005 (12)	−0.0013 (11)
C3	0.0360 (15)	0.0304 (13)	0.0339 (13)	−0.0053 (11)	−0.0040 (11)	−0.0005 (10)
C4	0.0382 (15)	0.0346 (13)	0.0387 (14)	−0.0050 (11)	−0.0031 (12)	−0.0006 (11)
C5	0.0510 (18)	0.0526 (17)	0.0391 (14)	−0.0094 (14)	0.0046 (13)	−0.0014 (12)
C6	0.065 (2)	0.0529 (18)	0.0436 (16)	−0.0102 (15)	−0.0023 (14)	0.0158 (13)
C7	0.062 (2)	0.0355 (15)	0.0610 (19)	−0.0001 (14)	−0.0046 (16)	0.0089 (13)
C8	0.0536 (18)	0.0362 (15)	0.0479 (15)	0.0007 (13)	0.0037 (13)	−0.0032 (12)
C9	0.0424 (16)	0.0402 (14)	0.0331 (13)	−0.0059 (12)	0.0014 (11)	−0.0041 (11)
C10	0.0360 (15)	0.0332 (13)	0.0294 (12)	−0.0059 (11)	−0.0029 (10)	0.0006 (10)
C11	0.0479 (17)	0.0374 (14)	0.0386 (14)	−0.0008 (12)	−0.0017 (12)	−0.0028 (11)
C12	0.0556 (19)	0.0400 (15)	0.0492 (16)	−0.0053 (14)	−0.0078 (14)	0.0067 (13)
C13	0.067 (2)	0.0562 (18)	0.0398 (16)	−0.0159 (16)	−0.0051 (14)	0.0147 (14)

### Geometric parameters (Å, °)

**Table:**

S1—C1	1.657 (2)	C4—H4	0.9300
Cl1—C9	1.741 (2)	C5—C6	1.378 (3)
N1—C2	1.382 (3)	C5—H5	0.9300
N1—C1	1.390 (3)	C6—C7	1.374 (4)
N1—H1	0.8600	C6—H6	0.9300
N2—C1	1.340 (3)	C7—C8	1.383 (3)
N2—C10	1.416 (3)	C7—H7	0.9300
N2—H2	0.8600	C8—H8	0.9300
N3—C9	1.316 (3)	C9—C10	1.384 (3)
N3—C13	1.332 (3)	C10—C11	1.377 (3)
O1—C2	1.218 (3)	C11—C12	1.379 (3)
C2—C3	1.480 (3)	C11—H11	0.9300
C3—C4	1.382 (3)	C12—C13	1.373 (4)
C3—C8	1.395 (3)	C12—H12	0.9300
C4—C5	1.378 (3)	C13—H13	0.9300
			
C2—N1—C1	128.66 (18)	C7—C6—H6	120.0
C2—N1—H1	115.7	C5—C6—H6	120.0
C1—N1—H1	115.7	C6—C7—C8	120.5 (2)
C1—N2—C10	125.59 (19)	C6—C7—H7	119.8
C1—N2—H2	117.2	C8—C7—H7	119.8
C10—N2—H2	117.2	C7—C8—C3	119.5 (2)
C9—N3—C13	116.4 (2)	C7—C8—H8	120.3
N2—C1—N1	114.80 (19)	C3—C8—H8	120.3
N2—C1—S1	125.53 (17)	N3—C9—C10	124.9 (2)
N1—C1—S1	119.66 (15)	N3—C9—Cl1	116.11 (19)
O1—C2—N1	121.9 (2)	C10—C9—Cl1	119.02 (18)
O1—C2—C3	122.4 (2)	C11—C10—C9	117.4 (2)
N1—C2—C3	115.72 (19)	C11—C10—N2	122.3 (2)
C4—C3—C8	119.5 (2)	C9—C10—N2	120.2 (2)
C4—C3—C2	122.2 (2)	C10—C11—C12	119.0 (2)
C8—C3—C2	118.3 (2)	C10—C11—H11	120.5
C5—C4—C3	120.4 (2)	C12—C11—H11	120.5
C5—C4—H4	119.8	C13—C12—C11	118.3 (2)
C3—C4—H4	119.8	C13—C12—H12	120.8
C6—C5—C4	120.0 (2)	C11—C12—H12	120.8
C6—C5—H5	120.0	N3—C13—C12	123.9 (2)
C4—C5—H5	120.0	N3—C13—H13	118.0
C7—C6—C5	120.1 (2)	C12—C13—H13	118.0
			
C10—N2—C1—N1	−175.8 (2)	C4—C3—C8—C7	1.8 (4)
C10—N2—C1—S1	5.3 (3)	C2—C3—C8—C7	−179.7 (2)
C2—N1—C1—N2	−8.8 (4)	C13—N3—C9—C10	0.8 (4)
C2—N1—C1—S1	170.2 (2)	C13—N3—C9—Cl1	−178.04 (19)
C1—N1—C2—O1	−3.9 (4)	N3—C9—C10—C11	−2.1 (4)
C1—N1—C2—C3	176.3 (2)	Cl1—C9—C10—C11	176.72 (18)
O1—C2—C3—C4	144.0 (3)	N3—C9—C10—N2	−178.1 (2)
N1—C2—C3—C4	−36.2 (3)	Cl1—C9—C10—N2	0.7 (3)
O1—C2—C3—C8	−34.6 (4)	C1—N2—C10—C11	50.7 (3)
N1—C2—C3—C8	145.3 (2)	C1—N2—C10—C9	−133.5 (2)
C8—C3—C4—C5	0.7 (4)	C9—C10—C11—C12	2.0 (3)
C2—C3—C4—C5	−177.8 (2)	N2—C10—C11—C12	177.9 (2)
C3—C4—C5—C6	−2.2 (4)	C10—C11—C12—C13	−0.8 (4)
C4—C5—C6—C7	1.2 (4)	C9—N3—C13—C12	0.6 (4)
C5—C6—C7—C8	1.2 (4)	C11—C12—C13—N3	−0.6 (4)
C6—C7—C8—C3	−2.7 (4)		

### Hydrogen-bond geometry (Å, °)

**Table:**

*D*—H···*A*	*D*—H	H···*A*	*D*···*A*	*D*—H···*A*
N2—H2···O1	0.86	1.94	2.633 (2)	137
N1—H1···S1^i^	0.86	2.74	3.5982 (18)	178
C12—H12···O1^ii^	0.93	2.70	3.324 (3)	125

Symmetry codes: (i) −*x*+1, −*y*+1, −*z*+1; (ii) −*x*+2, *y*−1/2, −*z*+3/2.
